# Influence of Leaf Age on the Scaling Relationships of Lamina Mass vs. Area

**DOI:** 10.3389/fpls.2022.860206

**Published:** 2022-04-08

**Authors:** Yabing Jiao, Karl J. Niklas, Lin Wang, Kexin Yu, Yirong Li, Peijian Shi

**Affiliations:** ^1^College of Biology and the Environment, Bamboo Research Institution, Nanjing Forestry University, Nanjing, China; ^2^Plant Biology Section, School of Integrative Plant Science, Cornell University, Ithaca, NY, United States

**Keywords:** diminishing returns, leaf development, leaf functional traits, LMA, resources utilization strategy

## Abstract

Leaf lamina mass and area are closely correlated with the photosynthetic capacity and competitive ability of plants, whereas leaf age has been demonstrated to affect physiological processes such as photosynthesis. However, it remains unknown whether the lamina mass vs. area scaling relationship is influenced by leaf age, which is important for understanding plant adaptive strategies and, more broadly, resource utilization and growth. We measured the leaf functional traits of five leaf-age groups of *Photinia* × *fraseri* for a total of 1,736 leaves. ANOVA followed by Tukey’s honestly significant difference test was used to compare the functional traits among the five leaf-age groups. Reduced major axis regression protocols were used to fit the scaling relationship between lamina mass and area, and the bootstrap percentile method was used to compare the lamina mass vs. area scaling relationships among the leaf-age groups. Lamina area, and the ratio of lamina dry mass to lamina fresh mass increased with increasing leaf age. Lamina fresh mass per unit area, and lamina dry mass per unit area both exhibited a parabolic-like trend as leaf age increased, i.e., at the leaf maturation stage, it showed a slight but significant decline. The phenomenon called diminishing returns were confirmed by each of the five leaf-age groups, i.e., all of the numerical values of the scaling exponents of lamina mass vs. area were significantly greater than 1. There were significant differences in the scaling exponents of lamina mass vs. area for the leaves across different sampling times. The scaling exponents were lower at the early rapid growth stage, indicating a lower cost for increasing leaf area compared to the leaf maturation stage. These data are consistent with leaves undergoing a transition from resource acquisition to resource conservation in the process of their development and growth.

## Introduction

Photosynthesis plays an indispensable and critical role in maintaining the balance of carbon and oxygen in the atmosphere. Leaves provide the most important albeit not the only organ for photosynthesis. Consequently, the functional traits of leaves are of primary interest in understanding plant biology and ecosystem dynamics. In the context of the functional traits, the leaf lamina mass and area are important indexes to describe photosynthetic capacity as well as reflecting the trade-off between the investment (“cost”) of leaf construction and photosynthetic return. Prior studies have shown that there is a significant allometric relationship between the lamina mass and area (Milla and Reich, 2007; Niklas et al., 2007; Huang et al., 2019; Liu et al., 2020) indicating that increases in the dry mass investment in lamina construction do not obtain a proportional increase in lamina area. This phenomenon has been described as diminishing returns (Niklas et al., 2007).

In addition to leaf lamina mass and area, the leaf lamina dry mass per unit area (LMA) is an important functional trait (Westoby et al., 2002; Poorter et al., 2009). Wright et al. (2004) concluded that the maximum photosynthetic capacity of leaves decreased significantly with increasing LMA such that species with faster resource utilization and return efficiency tend to have lower LMA, higher photosynthetic efficiency, and shorter leaf life spans. In a similar vein, some investigators (Niinemets, 2001; Westoby et al., 2002; Hikosaka and Shigeno, 2009) concluded that species with lower resource utilization rates and return efficiencies have high LMA, which is often used to reflect the adaptive abilities of plants to obtain carbon resources in different environments (Poorter et al., 2009; Cui et al., 2020). Therefore, the study of leaf functional traits can effectively reflect the photosynthetic capacity and competitiveness of plants.

Leaf age and leaf growth stages are crucial factors in morphological construction and physiological processes of many plants. For example, Niklas (1991) showed that the total leaf mass and total leaf area per plant are positively correlated with leaf age by comparing shoots with young and mature leaves of *Populus tremuloides* Michx. Mediavilla et al. (2014) contended that the interspecific differences in leaf longevity, morphology, and chemical composition of mature leaves are more notable compared to immature leaves. Ji et al. (2021) report that LMA and leaf dry matter content increase significantly during the development of leaves. In addition, previous studies have shown that leaf age is closely associated with photosynthetic capacity (Field and Mooney, 1983; Han et al., 2008). However, with increasing leaf age, photosynthetic capacity is known to gradually decrease (Kitajima et al., 1997). For example, Day et al. (2001) concluded that the leaves of the red spruce (*Picea rubens* Sarg.) manifest age-related trends in leaf morphology and physiology and that the decline in the productivity of old red spruce results from the age-related decline in photosynthetic rate.

**FIGURE 1:**
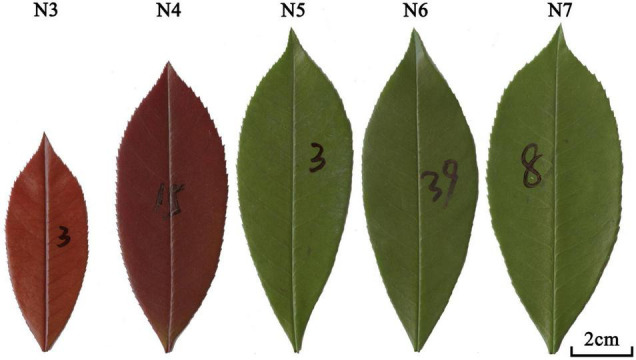
Examples of leaves from the five leaf-age groups. Newly developed leaves in early March 2021 were harvested in the middle of March, April, May, June, and July 2021, which were denoted by N3-7, respectively. The numbers on written on leaves were used to mark leaves harvested in different months.

However, prior research on the effects of leaf age on growth has mainly focused on conifers, e.g., *Pinus pinaster* Ait., *Pinus koraiensis* Sieb. et Zucc., and *Picea rubens* Sarg (Eimil-Fraga et al., 2015; Ji et al., 2021). To address this bias, we examined the shade-tolerant evergreen shrub species *Photinia* × *fraseri* to determine the effects of leaf age on leaf functional traits and the leaf (lamina) mass vs. area scaling relationship Specifically, we addressed the following two questions: (i) Do leaf functional traits change with leaf age? and (ii) Does the scaling relationship between leaf mass and area change with age? *Photinia* × *fraseri* is a hybrid between *Photinia glabra* and *Photinia serratifolia*. It is a nothospecies in the rose family, Rosaceae. *Photinia* × *fraseri* was selected for study because of its availability and because its evergreen leaves have a comparatively simple ovoid morphology, which makes measurements of lamina mass and area comparatively simple to make (Figure 1).

## Materials and Methods

### Leaf Collection Protocols

The sampling site was located at the campus of Nanjing Forestry University (118°48′35′′ E, 32°04′67′′ N), Nanjing, Jiangsu Province, China, which has a subtropical monsoon climate. The mean annual precipitation is 1,156 mm, and the mean annual temperature is 15.6°C based on the climate data collected between 1951 and 2014.^1^ The rainy season is from June to August, approximately accounting for half of the annual accumulated precipitation.

A total of 1,736 leaves were collected from 40-50 *Photinia* × *fraseri “*Red Robin” plants in 2021. Newly developed leaves in early March 2021 were collected in the middle of March, April, May, June, and July 2021. These collections are labeled as N3, N4, N5, N6, and N7, respectively (to denote “new” in each month) (Figure 1 and Table 1).

**TABLE 1:** Leaf collection information for *Photinia* × *fraseri* “Red Robin”.

Leaf-age group	Sampling date	Sample size	Leaf-unfolding time
N3	March 15, 2021	387	Early March 2021
N4	April 11, 2021	333	Early March 2021
N5	May 16, 2021	330	Early March 2021
N6	June 15, 2021	350	Early March 2021
N7	July 17, 2021	336	Early March 2021

### Measures of Leaf Functional Traits

We measured the fresh mass of each lamina using an electronic balance (Type: ML 204; Mettler Toledo Company, Greifensee, Switzerland; measurement accuracy 0.0001 g) and scanned the leaves to bitmap images at a 600-dpi resolution using a photo scanner (V550, Epson Indonesia, Batam, Indonesia). Adobe Photoshop (CS6, version: 13.0) was used to obtain a black and white image of each lamina. The M-file based on MatLab (version ≥ 2009a) developed by Shi et al. (2018) was used to extract the planar coordinates of each lamina. And then leaf *A* was calculated using an R script (version 4.0.3; R Core Team, 2020) developed by Shi et al. (2018) and Su et al. (2019). The fresh leaves were then dried to constant weight in an oven (Type: XMTD-8222; Jinghong Experimental Equipment Co., Ltd., Shanghai, China) at 80°C for 48 h to measure dry mass using the same electronic balance.

### Statistical Methods

We used the analysis of variance (ANOVA) with a 0.05 significance level to test whether leaf age had a significant effect on the leaf lamina area, the ratio of lamina dry mass to lamina fresh mass (i.e., LDM/LFM), the lamina fresh mass per unit area (LFMA), and the lamina dry mass per unit area (LMA). Provided that a significant effect of leaf area on each of the measures was found, we used Tukey’s honestly significance difference test with a significance level of 0.05 (Hsu, 1996) to test the significance of the difference between any two groups in the leaf lamina area, LDM/LFM, LFMA, and LMA. Additionally, we used a power-law function to fit the scaling relationship between any two variables of interest:


$$Y_{1}=\beta Y_{2}^{\alpha },$$

where *Y*_1_ and *Y*_2_ represent the two interdependent variables, respectively, and α and β are the scaling exponent and the normalization constant, respectively. In order to stabilize the variance of leaf measures, both sides of the equation were log-transformed (Niklas, 1994; Niklas et al., 2007):


y=γ+αx.

where *y* = ln *Y*_1_, *x* = ln *Y*_2_, and γ = ln β. The numerical values of α and γ were obtained using reduced major axis regression protocols (Niklas, 1994; Smith, 2009). The bootstrap percentile method (Efron and Tibshirani, 1993; Sandhu et al., 2011) was used to compare the significance of the differences of the numerical values of α between any two leaf-age groups. For any two leaf-age groups (denoted as groups A and B), we calculated 4,000 replicates of α for each group using the bootstrap method (Efron and Tibshirani, 1993). Denoting D as the differences in the replicates of α between groups A and B, we observed whether the 95% CI of D included 0. If the lower bound of the 95% CI of D is larger than 0, it indicates that the estimated α-value of group A is larger than that of group B; if the upper bound of the 95% CI of D is smaller than 0, it indicates that the estimated α-value of group A is smaller than that of group B; if the 95% CI of D includes 0, it indicates that there is no significant difference in the estimated α-values between groups A and B (see Sandhu et al., 2011 for details). To measure the goodness of fit, the root-mean-square error (RMSE = the square root of the quotient of residual sum of squares and sample size) was used. All statistical analyses were carried out using R (version 4.0.3; R Core Team, 2020).

## Results

**FIGURE 2:**
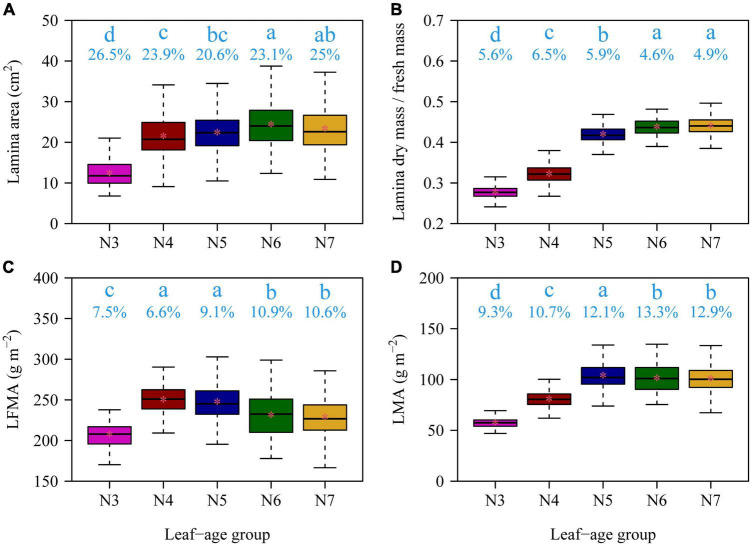
Boxplots of lamina area **(A)**, the ratio of lamina dry mass to lamina fresh mass **(B)**, lamina fresh mass per unit area **(C)**, and lamina dry mass per unit area **(D)** for the five leaf-age groups. The lowercase letters indicate the significance of the difference between any two of the five groups; the numbers below the lowercase letters are the coefficients of variation. In each panel, there was a significant difference (*p* ≤ 0.05) between any two leaf-age groups that did not share the same letter, and there was no significant difference (*p* > 0.05) between any two leaf-age groups that shared the same letter based on Tukey’s HST test with the 0.05 significance level.

**FIGURE 3:**
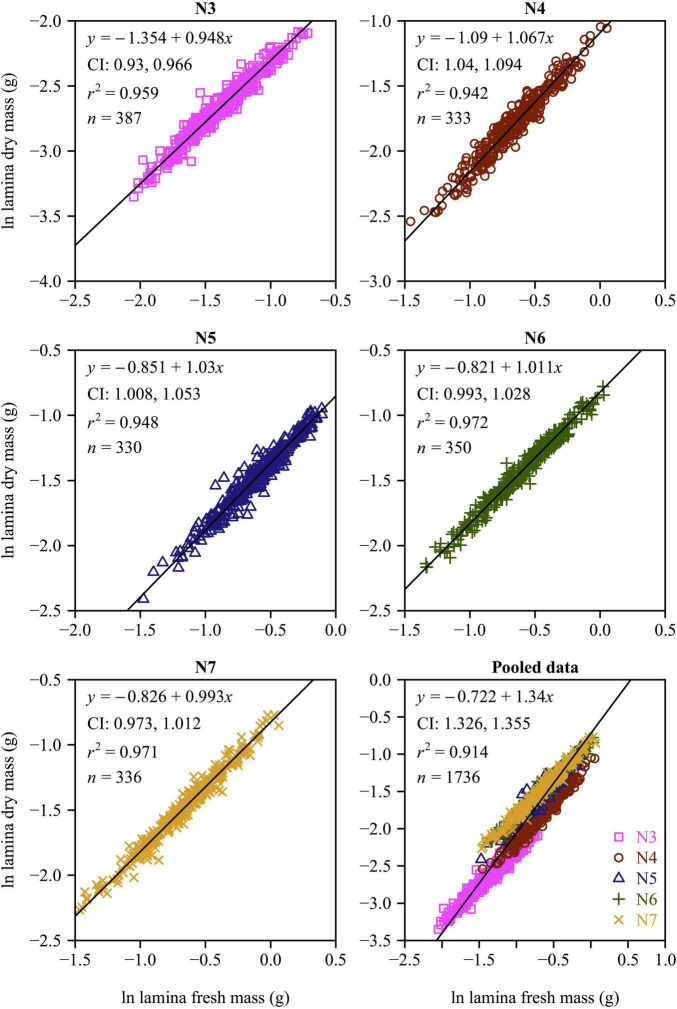
Fitted scaling relationships between lamina dry mass and lamina fresh mass for the five leaf-age groups. The small open circles represent log-transformed values of lamina dry mass vs. lamina fresh mass; the red straight lines represent the regression lines; CI represents the 95% confidence intervals of the slope; *r*^2^ is the coefficient of determination; and *n* is the sample size.

**FIGURE 4:**
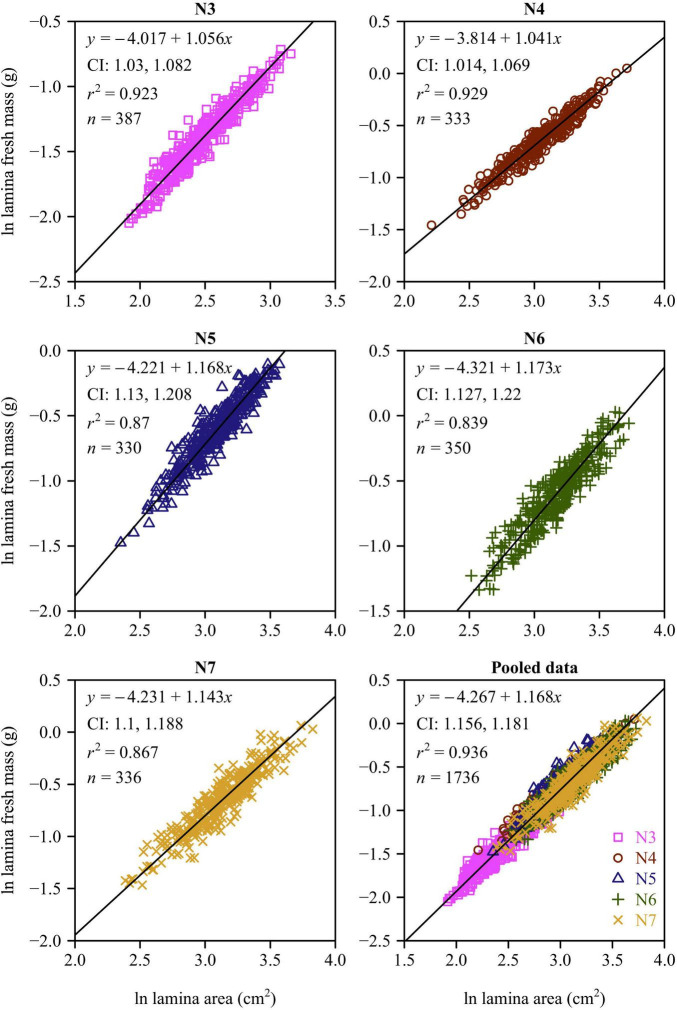
Fitted scaling relationships between lamina fresh mass and lamina surface area for the five leaf-age groups. The small open circles represent the log-transformed values of lamina fresh mass vs. lamina surface area; the red straight lines represent the regression lines; CI represents the 95% confidence intervals of the slope; *r*^2^ is the coefficient of determination; and *n* is the sample size.

**FIGURE 5:**
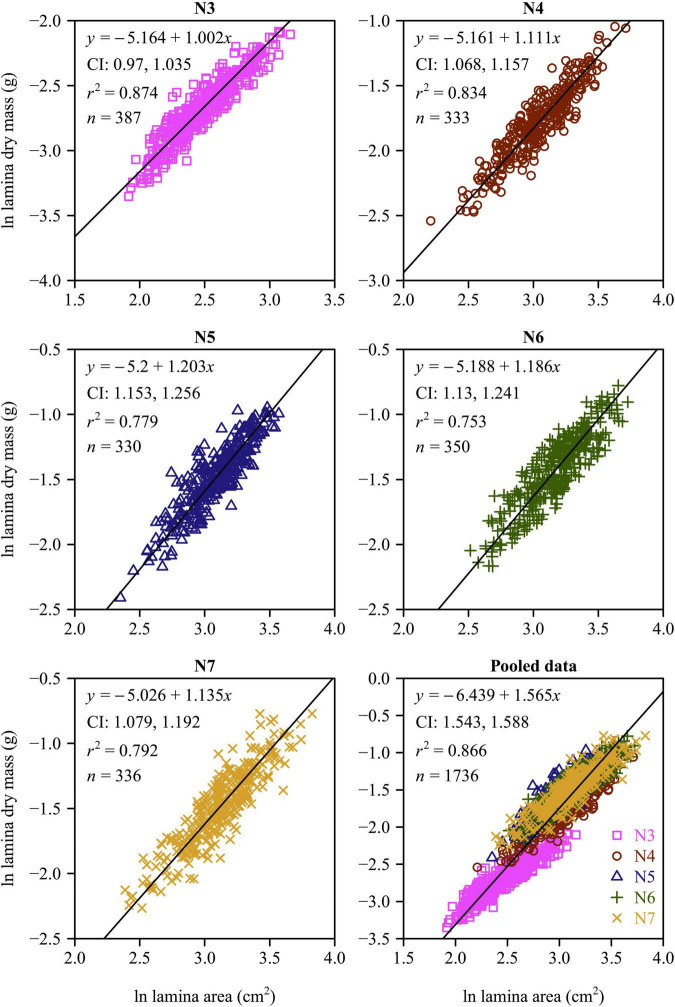
Fitted scaling relationships between lamina dry mass and lamina surface area for the five leaf-age groups. The small open circles represent the log-transformed values of lamina dry mass vs. lamina surface area; the red straight lines represent the regression lines; CI represents the 95% confidence intervals of the slope; *r*^2^ is the coefficient of determination; and *n* is the sample size.

There were significant effects of leaf age on the leaf lamina area, LDM/LFM, LFMA, and LMA among the five leaf-age groups (all *p*-values < 0.01). With increasing leaf age, both the lamina area and LDM/LFM significantly increased (Figure 2). The LMA and LFMA both exhibited a parabolic-like trend with increasing age, i.e., LMA and LFMA increased and then declined with increasing leaf age (Figure 2). All of the functional leaf traits examined over the course of this study were significantly correlated within each of the five leaf-age groups when the data were log-log transformed (Figures 3–5). Each of the coefficients of determination (*r*^2^) equaled or exceeded 0.75 for each of the leaf-age groups. There was a statistically significant log-log linear relationship between LDM and LFM for each leaf-age group. The 95% confidence intervals for the N6 and N7 included unity, and the lower bound of the 95% confidence intervals of the scaling exponents of LDM vs. LFM for N5 was approximately equal to 1 with a difference from 1 < 0.01. For N3 and N4, the difference between the upper bound of the 95 CIs of the slope and 1 and between the lower bound of the 95 CIs of the slope and 1 were slightly larger, with the absolute value of 0.04. This indicated that the mature leaves maintained an isometric scaling relationship, whereas the young leaves tended to slightly deviate from the isometric scaling. However, for the pooled data, the scaling exponent of LDM vs. LFM was overestimated because of the differences in water content across the different leaf-age groups (Figure 3).

There was a strong positive relationship between lamina mass and area for each leaf-age group (Figures 4, 5). The lower bound of the 95% CIs of the lamina mass vs. area scaling relationship exceeded unity for each of the five leaf-age groups, indicating that the lamina mass vs. area scaling relationship is allometric. For each leaf-age group, the scaling exponents of LFM vs. lamina area and LDM vs. lamina area exceeded unity, and were therefore consistent with the hypothesis of “diminishing returns.” However, the *r*^2^ value of the LDM vs. lamina area scaling relationship was lower than that of the LFM vs. lamina area scaling relationship for each leaf-age group (compare Figure 4 with Figure 5). In addition, for the pooled data, the scaling exponent of LDM vs. lamina area was largely overestimated in comparison with that of LFM vs. lamina area.

**FIGURE 6:**
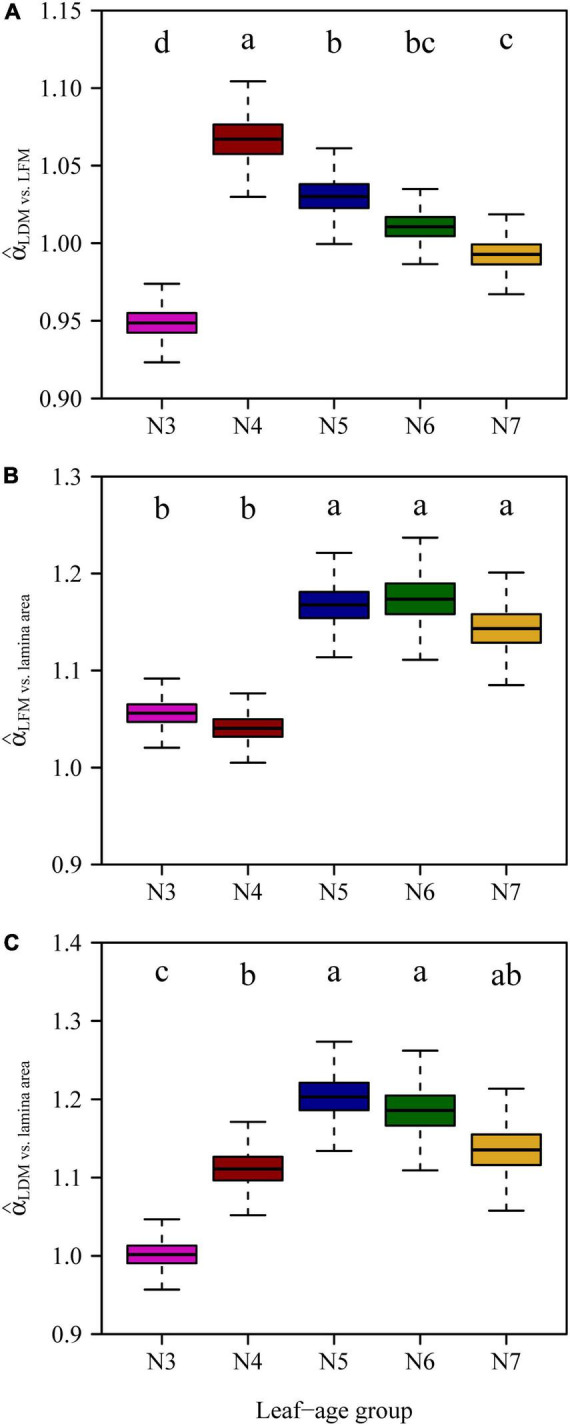
Comparisons of the scaling exponents of LDM vs. LFM **(A)**, LFM vs. lamina area **(B)**, LDM vs. lamina area **(C)** for the five leaf-age groups. The lowercase letters indicate the significance of the difference between any two of the five leaf-age groups.

With the exception of N3, the numerical values of the LDM vs. LFM scaling exponents decreased and converged onto a value of 1 with increasing leaf age (Figure 6). The scaling exponents of leaf *M* vs. lamina area for N3 and N4 were significantly smaller than those of N5, N6 and N7 (Figure 6), indicating that older leaves had larger costs of construction.

## Discussion

### Variations in Leaf Functional Traits Across Leaf-Age Groups

Prior research has shown that the photosynthetic capacity of leaves decreases with increasing leaf age (Horsley and Gottschalk, 1993; Bauerle et al., 2020), and that this might be associated with the changes in chemical composition, leaf nitrogen content, and CO_2_ diffusion limitation (Zhang et al., 2008). Photosynthetic rates of fully expanded leaves generally show a decline with increasing leaf age due to the reallocation of resources to young leaves to optimize the overall systemic photosynthetic returns of an individual plant, rather than resulting from leaf functional degradation (Field and Mooney, 1983; Hikosaka et al., 1994). Moreover, the photosynthetic capacity of young leaves has been shown to be significantly lower than mature leaves (Hikosaka and Shigeno, 2009; Liu et al., 2020; Ji et al., 2021). Although leaf functional traits have been regarded as important references for understanding plant ecological and growth strategies (Westoby et al., 2000, 2002), there are trade-offs among different leaf functional traits (e.g., lamina area, LMA) under the limitation of resources. If plants increase their investment to one functional trait, they are likely to reduce their investment to other functional traits (Falster et al., 2018).

The data presented here show that the leaf lamina area, the ratio of LDM to LFM, and LMA generally increase with increasing leaf age, which is consistent with previous reports in other species (Niklas, 1991; He and Yan, 2018; Liu et al., 2020; Guo et al., 2021). In addition, LFMA decreased at the leaf maturation stage. There are two interrelated explanations for this phenomenology. First, over the course of leaf development, maturation, and subsequent senescence, the dry matter investment disproportionately increases with increasing age, and, second, the symplastic volume fraction (the living contents of a leaf, i.e., the protoplasmic contents) systematically decreases with increasing age. Both of these trends are reflected in the trends of lamina area, LDM/LFM, LFMA, and LMA reported here with increasing leaf age, and both are clearly and intrinsically interrelated, although the proximate mechanism(s) underlying each of these trends differs. During leaf expansion, leaves generally have a low cost of construction with increasing leaf area because leaf expansion involves the volumetric increase in cell size (and increase in the symplastic volume fraction) rather than the addition of significant amounts of cell wall (apoplastic) materials. The reverse is generally true during leaf maturation during which the apoplastic volume fraction increases relative to the symplasic volume fraction as a consequence of the deposition of secondary cell wall materials including the lignification of vascular tissues. This general phenomenology is consistent with the trends in our data (see Figure 2).

The aforementioned growth dynamic has obvious physiological and biomechanical consequences. For example, species with long versus short leaf longevities must adopt specific and different strategies to maintain growth (Mediavilla et al., 2014). It is not surprising therefore that previous studies have shown that variations in LMA are correlated across plant functional groups (such as trees, shrubs, herbs) as well as abiotic variables such as light, temperature, water and nutrient availability, and atmospheric composition (Ackerly, 1992; Poorter and de Jong, 1999; Niinemets, 2001; Poorter et al., 2009). In our study site, ambient temperature and precipitation gradually increase during the rainy season, especially in June, July, and August, over the course of leaf initiation, expansion, and maturation (see the online Supplementary Material in Li et al., 2022). LMA has been demonstrated to have plastic responses to climate, e.g., in arid environments plants can reduce water requirements, which results in a higher LMA relative to those in humid environments (Poorter et al., 2009). Similarly, at the leaf maturation stage, the ratio of lamina dry mass to fresh mass reached a stable status, and the precipitation might play an important role in affecting the numerical value of LMA. For example, June and July receive greater amount of rain than May, which perhaps to a certain degree accounts for why the LMAs in June and July are significantly but slightly lower than that in May (Figure 2). Thus, changing but predictable environmental conditions might have exerted an influence to a certain degree on the expression of leaf functional traits (Poorter et al., 2009). Nevertheless, it requires additional controlled experiments for testing whether the amount of precipitation can change LMA, i.e., a plasticity of LMA to weather, in future studies.

### Influence of Leaf Age on Leaf Allometry

The scaling exponents of LDM vs. LFM were found to be significantly different between March and April, likely because new leaves were growing expanding at this time. Figure 2 shows significant increases in both the lamina area and LDM/LFM between March and April. With the exception of March, the scaling exponents of LDM vs. LFM decreased toward 1 with increasing leaf age (Figure 6). We interpret these data to indicate that leaf water content and leaf dry mass tend to synchronously increase.

Liu et al. (2020) confirmed that for leaves of *Alangium chinense* (Lour.) Harm and *Liquidambar formosana* Hance, the scaling exponents of the lamina mass vs. area scaling relationship during the spring are significantly higher than during the summer. This is consistent with our results. In the case of N3 and N4, the numerical values of the lamina mass vs. area scaling exponents were significantly smaller compared to those for N5, N6, and N7 (Figure 6), indicating that the larger leaf area of mature leaves require greater apoplastic investments. Under any circumstances, it is obvious that the investment in leaf construction changed between the early rapid growth and leaf maturation stages. Changes in leaf investment may be related to photosynthetic returns (Day et al., 2001; Bielczynski et al., 2017), such that young leaves produce less photosynthates (Falster and Westoby, 2003) compared to more mature leaves (Pan et al., 2012).

The numerical values of the scaling exponents of the lamina mass vs. area scaling relationship for each of leaf-age groups were in excess of unity, and thus conform with the phenomenon called “diminishing returns” (see also Huang et al., 2019; Liu et al., 2020). In general, although larger leaf lamina area can yield a higher photosynthetic capacity, progressively larger leaves generally require a disproportionately greater investment in their mechanical support (Niklas, 1992, 1999; see also Gibert et al., 2016). This disproportionate investment in the apoplastic content relative to the symplastic content per unit leaf area can account for the numerical values of the lamina mass vs. area scaling relationship approaching and exceeding unity (i.e., α> 1.0).

## Conclusion

Our data indicate that both lamina area and ratio of lamina dry mass to lamina fresh mass increase with increasing leaf age, whereas LMA and LFMA manifest a parabolic-like trend. With the exception of leaves collected in March, the numerical values of the LDM vs. LFM scaling exponents decreased and converged onto 1, indicating a proportional (isometric) relationship between leaf dry mass and leaf absolute water content. All of the numerical values of the lamina mass vs. area scaling exponents exceeded 1, thereby confirming the phenomenon called diminishing returns. Based on these numerical values and the trends exhibited, we concluded that the costs of leaf construction increase and subsequently plateau as a consequence of an increase in the apoplastic volume fraction per unit leaf area over the course of leaf expansion, maturation, and early senescence over the course of leaf ontogeny. We believe that this ontogenetic pattern holds for the leaves of all plant species. Future research is necessary, however, to validate this speculation, and needs to be expanded to include the leaves of different functional and phyletic plant groups (e.g., ferns, cycads, and monocots).

## Data Availability Statement

The original contributions presented in the study are included in the article/Supplementary Material, further inquiries can be directed to the corresponding authors.

## Author Contributions

KN and PS designed this work, analyzed the data, and revised the manuscript. YJ, LW, KY, and YL carried out the experiment. YJ wrote the initial draft. All authors commented on and agreed with this submission.

## Conflict of Interest

The authors declare that the research was conducted in the absence of any commercial or financial relationships that could be construed as a potential conflict of interest.

## Publisher’s Note

All claims expressed in this article are solely those of the authors and do not necessarily represent those of their affiliated organizations, or those of the publisher, the editors and the reviewers. Any product that may be evaluated in this article, or claim that may be made by its manufacturer, is not guaranteed or endorsed by the publisher.

## Footnotes

1
http://data.cma.cn
